# High-order chromatin structures mediated by phase separation in cell senescence

**DOI:** 10.3389/fmolb.2026.1896884

**Published:** 2026-09-28

**Authors:** Wenjing Sun, Boxian Li, Zhaoyong Xu, Jing Wang, Jing Cao, Yangzhong Wang, Jiale Wang, Wenbo Gu, Pengjun Xu, Liang Wang, Wenyan Xie

**Affiliations:** 1 Technology Center of Shanghai Tobacco Group Co., Ltd., Shanghai, China; 2 Key Laboratory of Pesticide and Chemical Biology of Ministry of Education, Hubei Key Laboratory of Genetic Regulation and Integrative Biology, School of Life Sciences, Central China Normal University, Wuhan, China; 3 Tobacco Research Institute, The Chinese Academy of Agricultural Sciences, Qingdao, China

**Keywords:** 3D genome architecture, biomolecular condensates, cellular senescence, chromatin compartmentalization, condensatopathies, epigenetics, liquid-liquid phase separation (LLPS), nucleolus

## Abstract

Liquid-liquid phase separation (LLPS) coordinates biomolecular condensates to establish three-dimensional chromatin architecture and epigenetic regulation. The progressive loss of epigenetic information is a fundamental, reversible driver of cellular senescence. In this review, we systematically synthesize emerging evidence on the spatiotemporal dynamics of LLPS-mediated chromatin compartmentalization during the aging process. We highlight how age-dependent alterations in histone, RNA, and DNA modifications disrupt multivalent interactions, leading to constitutive heterochromatin dissolution, aberrant transcriptional condensate formation, and nucleolar expansion. Furthermore, biomolecular condensates undergo age-dependent biophysical maturation—shifting from dynamic liquid states to solid-like aggregates—which dictates structural chromatin erosion and topologically associating domain (TAD) fusion. These diverse biophysical defects can be consolidated within a unified “condensatopathy” framework, encompassing both loss-of-function failures and toxic gain-of-function protein aggregations across somatic aging, stem cell exhaustion, and neurodegeneration. Understanding how phase separation regulates higher-order chromatin structures provides profound insights into aging trajectories. Ultimately, selectively modulating the material properties of biomolecular condensates, rather than merely dissolving them, offers a transformative precision therapeutic paradigm to restore epigenetic fidelity and counteract age-related diseases.

## 1 Introduction

Aging is mainly divided into two categories: physiological aging and pathological aging. The former refers to age-dependent physiological degradation processes after maturity, such as organ aging, weakness, and increased susceptibility to diseases. The latter is caused by external factors, such as neurodegenerative diseases, immune response weakness, malnutrition, metabolic disorders, etc. These two aging processes are difficult to distinguish and are often interrelated (Lopez-Otin et al., 2013). In recent years, there has been significant progress in aging research, especially in the conserved epigenetic pathways (Sen et al., 2016; Zhang et al., 2020; An et al., 2025). However, due to the complexity of their causes, many outstanding questions remain unclear, and delaying or reversing the aging process is still a huge challenge.

The changes in transcription factors and chromatin status related to age are critical features of the aging process and important hubs connecting many hallmarks of aging processes. In mammals and reported aging models, there are global and local DNA methylation changes in the genome; loss of histones and histone post-translational modifications (PTMs); chromatin remodeling; RNA modification, and non-coding RNA (ncRNA) regulation (Sen et al., 2016; Zhang et al., 2020; Wang et al., 2022). The epigenetic changes further affect the higher-order chromosomal structure, leading to the shift on chromatin compartmentalization linked with longevity and cancers (Ma and Sen, 2026). Distinct compartments are enriched with specific PTMs, and cognate “readers” and “writers” of these marks accumulate as biomolecular condensates formed via LLPS mediated by multivalent interactions (Wang et al., 2019; Gibson et al., 2019; Sun et al., 2025; Xia et al., 2025; Rajshekar et al., 2025) (for general reviews of the LLPS concept, including cytosolic condensates such as stress granules, see (Banani et al., 2017; Shin and Brangwynne, 2017)). Furthermore, architectural chromatin proteins such as HMGA1 establish heterogeneous three-dimensional chromatin networks through phase separation-mediated compartmentalization, functioning as transcriptional buffers for inflammatory signaling; loss of HMGA1 drives SASP gene upregulation and promotes cellular senescence heterogeneity (Olan et al., 2024). In aging hematopoietic stem cells (HSCs), the RNA-binding protein (RBP) FUS undergoes aberrant phase transition that alters chromatin architecture and impairs stem cell function (Tang et al., 2024), establishing a direct link between RBP phase separation and age-dependent chromatin remodeling.

Other chromatin sub-compartments can also accumulate proteins with intrinsically disordered regions (IDRs). It is important to distinguish such phase-separated compartments from CTCF-independent loops and topologically associating domains (TADs): loops and TADs are architectural features generated mainly by cohesin-mediated loop extrusion, whereas sub-compartments are phase-separated chromatin compartments—two distinct modes of 3D genome organization. Recent studies have shown that cancer establishes oncogenic transcription factor condensates through LLPS and induces aberrant chromatin regulatory complexes and three-dimensional chromatin structures during tumor transformation (Ahn et al., 2021; Lu et al., 2021; Taghbalout et al., 2025). Nevertheless, the molecular underpinnings of aging-associated changes in epigenomic morphology including chromatin organization regulated by LLPS, remain poorly understood (Xie and Han, 2025; Gutierrez and Tyler, 2026). Intriguingly, recent evidence has revealed that biomolecular condensates undergo an aging or maturation process, during which their internal diffusion dynamics and material properties progressively change, adding another layer of complexity to the relationship between condensate biology and cellular aging (Deniz, 2025) (Figure 1). This condensate aging phenomenon may have profound implications for understanding how the temporal dynamics of phase-separated compartments contribute to age-dependent epigenetic alterations.

**FIGURE 1:**
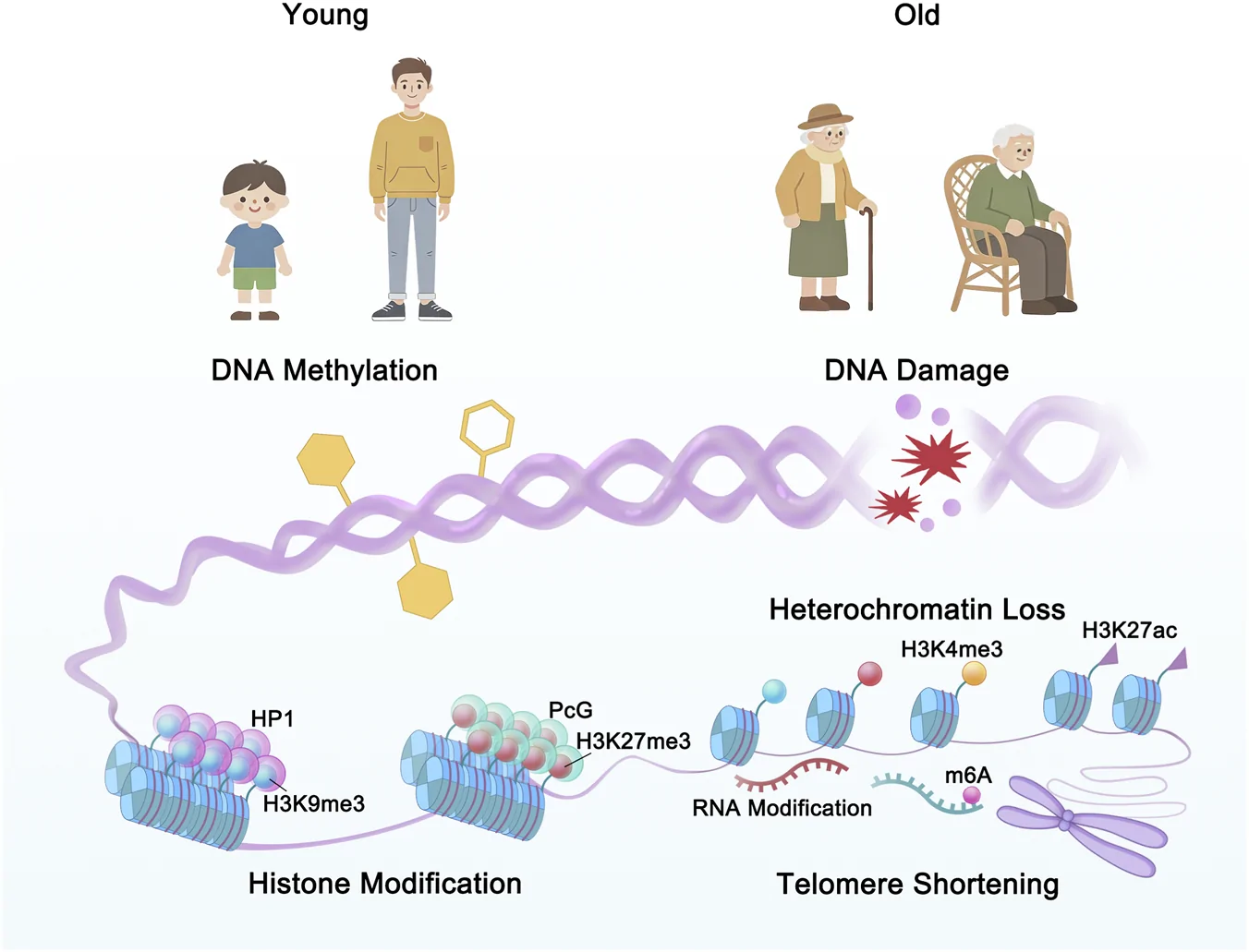
Epigenetic alterations and phase separation hallmarks in aging. Summary of aberrant phase separation mechanisms leading to aging through the dysregulation of post-translational modifications (PTMs) and a perspective model of phase-separated epigenetic hallmarks associated with the aging process.

In this review, we will focus on the role of chromatin compartmentalization regulated by phase separation in aging, providing key insights into age-dependent epigenomic changes during the onset and development of aging, and with the hope of developing new drugs to regulate abnormal biomolecular condensates, delay or even reverse aging and age-related diseases (Li et al., 2026; Qin et al., 2025).

## 2 Histone modification and phase separation in aging

Chromatin compartments contain specific histone/DNA covalent modifications that can be recognized by the corresponding reader proteins, and the two interact to cause a series of reactions, affecting the structure of chromatin and further gene expression (Xia et al., 2025) (Figure 1). Multivalent H3K9me3 and H3K27me3 marks, histone acetylation, and their multivalent readers mediate chromatin compartment formation through interaction-driven LLPS (Wang et al., 2019; Gibson et al., 2019; Sun et al., 2025; Uckelmann et al., 2025). Global change of post-translational modifications of histones can activate or silence gene expression and regulate the aging process; the methylation and acetylation of lysine residues are the most extensively studied (Malone et al., 2026; Hammonds et al., 2026).

### 2.1 Histone methylation

H3K4me3 is a marker associated with active transcription and plays an important role in determining aging and lifespan by regulating the expression of aging-related genes (Santos-Rosa et al., 2002). Aged mammalian stem cells have increased enrichment of H3K4me3 at intragenic cryptic promoter regions, the more permissive chromatin state likely underlies increased cryptic transcription (McCauley et al., 2021). SAGA-associated factor 29 (SGF29) is a recognition and binding module of H3K4me2/3, which forms liquid-like condensates in the nuclei of senescent cells (Yan et al., 2023). The formation of SGF29 condensates with its co-activators, such as MED4 and Pol II, and H3K4me3 binding, are both important for correct chromatin localization and gene activation (Cho and O'Farrell, 2023). These dynamic changes of chromatin organization thereby initiate gene expression related to cell senescence, including *CDKN1A*. Notably, the histone methyltransferase complex component MRG15 has also been found to form phase-separated condensates through its intrinsically disordered region, and MRG15 depletion accelerates cellular senescence in human mesenchymal stem cells, linking chromatin regulator phase separation directly to aging control (Li et al., 2025).

Gene silencing by chromatin compaction is integral to establishing and maintaining cell fates. H3K9me3-marked heterochromatin is mainly a constitutive type of chromatin and mediates chromatin compartmentalization through the multivalent PTM-reader interaction mediated by LLPS mechanism (Wang et al., 2019; Williams et al., 2024). Large-scale spatial rearrangement of chromatin, especially reorganization of H3K9me3 compartments in the nucleus, often accompanies the aging process (Lopes-Paciencia and Ferbeyre, 2025; Bensaha et al., 2025). The majority of oncogene-induced senescence (OIS) cells form a nuclear structure called senescence-associated heterochromatic foci (SAHF), inducing the activation of the tumor-suppressor pathways p53 and pRB/p16INK4a (Chandra et al., 2015). SAHF are enriched in constitutive heterochromatic markers, such as H3K9me3 and HP1 proteins (Narita et al., 2003). In contrast, the dissolution and deficiency of constitutive heterochromatin marked by H3K9me3, are typically observed in Hutchinson-Gilford progeria syndrome (HGPS) patients and replicative senescence. Sirtuin 3 (SIRT3) and SIRT7 are sirtuin family members implicated in aging and disease. SIRT3 and SIRT7 form a complex with nuclear lamina proteins and heterochromatin proteins, such as KAP1 and HP1 proteins, thus maintaining the repressive state of heterochromatin at the nuclear periphery (Bi et al., 2020; Diao et al., 2021). SIRT3/7 expression declines during human mesenchymal stem cell (hMSC) aging and SIRT3/7 deficiency accelerates senescence, which is partially through decreased heterochromatin stabilization.

Apolipoprotein E (APOE) is a component of lipoprotein particles. APOE cooperates with the autophagy-lysosomal machinery, including LC3 and the receptor p62/SQSTM1, to mediate the degradation of nuclear-envelope and heterochromatin-associated proteins, thereby destabilizing heterochromatin and driving senescence (Zhao et al., 2022). Complementary to the LLPS-based stabilization of heterochromatin discussed above, these studies reveal that heterochromatin integrity during aging is jointly guarded by condensate formation and selective protein turnover. H3K27me3-enriched facultative heterochromatin is distributed around the periphery of the H3K9me3 compartment. The Polycomb group (PcG) subunits form phase-separated condensates at H3K27me3 loci, which undergo facultative heterochromatin formation (Uckelmann et al., 2025; Niekamp et al., 2024; Gemeinhardt et al., 2025). The level of H3K27me3 determines the cell-type-specific roles in life span regulation; a well-known target of the PcG family during aging is *Cdkn2a* (encoding p16INK4a). CBX7, an H3K27me3 reader, can modulate lifespan and delay cellular senescence when ectopically expressed. A global loss of H3K27me3 in aged *C. elegans* and prematurely aged cells from HGPS, while in killifish and mouse brains, global H3K27me3 increases with age. The conserved histone lysine demethylases jmjd-1.2/PHF8 and jmjd-3.1/JMJD3 could work as positive regulators of lifespan in response to mitochondrial dysfunction (Wang et al., 2022). Moreover, an intrinsically disordered region contained in JMJD3 is involved in the formation of phase-separated biomolecular condensates, which are found in the enhancer cluster (Vicioso-Mantis et al., 2022). Ezh2 and PRC1 complex member CBX2 also form phase-separated condensates that contribute to facultative heterochromatin formation and regulate aging-related gene expression.

Beyond lysine methylation, histone arginine methylation has emerged as another critical regulator of chromatin stability and aging. Asymmetric dimethylation of histone H4 at arginine 3 (H4R3me2as), catalyzed by protein arginine methyltransferase 1 (PRMT1), is essential for maintaining histone H4 stability. During cellular senescence, reduced H4R3me2as levels lead to increased PA200 binding to H4, promoting proteasome-mediated H4 degradation independently of polyubiquitination, ultimately resulting in nucleosome disassembly and transcriptional activation of senescence-associated genes (Lin et al., 2020). Notably, the anti-aging compounds metformin, rapamycin, and resveratrol can restore H4 levels and counteract this degradation pathway, suggesting that histone arginine methylation represents a pharmacologically targetable node in the aging cascade. These findings establish PRMT1-mediated H4R3me2as as a guardian of nucleosome integrity and highlight how loss of arginine methylation can trigger a cascade of chromatin destabilization driving the senescent phenotype. LLPS plays an important role in maintaining chromatin compartmentalization and stabilizing the 3D genome, further discussion should be focused on the potential LLPS mechanism of reversing the aging process.

### 2.2 Histone acetylation

Histone acetylation is mediated by lysine acetyltransferases and is increased in active gene regions. In the presence of multi-bromodomain proteins (such as BRD4), new phase-separated droplets are formed after chromatin is highly acetylated by p300. These new droplets, which are enriched in acetylated chromatin, are immiscible with droplets of unmodified chromatin, similar to the aforementioned immiscible H3K9me3 and H3K27me3 compartments (Strom et al., 2024a; Strom et al., 2024b). In replicative senescent cells, p300 promotes the formation of active enhancer elements in the non-coding genome and significantly increases the levels of H3K122ac and H3K27ac at the proximal senescence-specific gene promoters. There are also substantial H3K27ac marks and BRD4 binding at promoters, which is confirmed to be a primary driver of the senescent phenotypes (Sen et al., 2019). A recent study shows that the intrinsically disordered regions, transcription factor-binding domains, and HAT activity of p300 all collectively contribute to biomolecular condensate formation by BRD4-p300 (Kosno et al., 2023). Aberrant BRD4-p300 condensates have been proposed to play a role in several cancers, and inflammation in atherosclerosis. Upregulation of p300/CBP also induces APP protein and beta-site APP cleaving enzyme 1 (BACE1) overexpression in Alzheimer’s disease. Depletion of p300 alone is sufficient to downregulate senescence genes and delay replicative senescence. Recent studies have further revealed that p300/CBP intrinsically disordered regions enable phase separation and condensate formation, and an IDR within CBP serves as an autoregulator of CBP KAT activity by buffering condensate composition and dynamics (Gilbert and Bose, 2026).

HDACs are considered to function as corepressors and, together with HATs, play a critical role in longevity. YY1 binds to HDAC1/3 and regulates METTL3 expression in a moderate LLPS manner (Li et al., 2022). Also, METTL3 is capable of self-interaction and likely provides the multivalency to drive condensation (Li et al., 2022). Lower levels of m6A RNA methylation and METTL3 were found in a premature aging human mesenchymal stem cell (hMSC) model. In oxidative-stress-induced cellular aging, the enhancement of METTL3 mediates m6A RNA modifications and facilitates the translation of the senescence marker p21. The reduced binding of HDAC2 in its promoter region can also trigger the upregulation of p21. METTL3-mediated m6A RNA also promotes age-related telomere shortening. Therefore, the link between histone acetylation and the control of RNA modification suggests that regulating its phase state is a potentially critical facet of its function in the aging process. The reciprocal interplay between acetylation writers, erasers, and RNA modification machinery operating within phase-separated compartments highlights the integrated nature of epigenetic regulation in aging.

### 2.3 Histone phosphorylation and ubiquitylation

Persistent DNA damage can block transcription and replication, thus hampering cellular functionality and promoting cellular senescence and apoptosis. The phosphorylation of a chromatin component, histone H2AX, was discovered as an integral part of the DNA damage response (DDR) in eukaryotes. Microscopically visible DDR foci, including 53BP1 and γ-H2AX, are dynamic and show droplet-like behavior (Levone et al., 2021; Li et al., 2024). The scaffolding protein AHNAK binds to the 53BP1 oligomerization domain and controls its multimerization potential. Loss of AHNAK results in hyper-accumulation of 53BP1 on chromatin and enhanced LLPS, culminating in an elevated p53 response, and leading to senescence in cells (Ghodke et al., 2021). In addition to DSB repair modulation, 53BP1 contributes to the maintenance of heterochromatin integrity and genome stability through LLPS with HP1α (Zhang et al., 2022). SUMOylated RNF168 undergoes LLPS, which restricts the recruitment of RNF168 to DNA damage sites, reduces RNF168-catalyzed H2A ubiquitination, restrains 53BP1 in nuclear condensates, and ultimately impairs nonhomologous DNA end joining repair efficiency (Wei et al., 2023).

DNA damage sites recruit RNA Polymerase II, which initiates transcription at the DSB site to generate damage-induced long non-coding RNAs (dilncRNAs). These RNAs can recruit RNA binding proteins (RBPs) such as FUS to drive LLPS. Upon DNA damage, the C-terminal RGG repeats of FUS form repair domains in response to PARP activity mediated by LLPS (Levone et al., 2021). Beyond its role in acute DNA repair, FUS phase separation has recently been implicated in aging-associated chromatin disruption. In aging hematopoietic stem cells, elevated FUS protein forms aberrant condensates that impair CTCF binding to chromatin, leading to TAD fusion and disruption of higher-order chromatin architecture, ultimately contributing to HSC functional decline (Tang et al., 2024). This finding establishes a direct mechanistic link between RBP phase transition, 3D genome restructuring, and stem cell aging. Bleomycin-induced cellular senescence also involves the accumulation of DNA damage and reorganization of the epigenome, characterized by KDM4-mediated demethylation of H3K9 and H3K36, leading to the transformation of high-order chromatin structure. The intersection of histone phosphorylation, ubiquitination, and protein phase separation in the DDR underscores the multifaceted role of PTMs in orchestrating both acute damage responses and long-term chromatin stability during the aging process.

Together, these findings highlight the diversity of phase separation-mediated chromatin regulatory mechanisms that contribute to cellular senescence (Table 1). From histone readers such as ΗP1α and PRC1-CBX that organize constitutive and facultative heterochromatin, to DNA methylation readers such as MeCP2 and RNA modification writers such as METTL3 that bridge epigenetic signaling with 3D chromatin architecture, a comprehensive picture emerges in which virtually every layer of epigenetic regulation converges on biomolecular condensate formation to modulate aging-associated transcriptional programs. The identification of architectural chromatin organizers such as HMGA1, together with the I-BAR-domain protein IRTKS—which has recently been shown to form heterochromatin-stabilizing condensates that counteract cellular senescence (Olan et al., 2024; Xie et al., 2024)—further extends this framework beyond traditional epigenetic marks to encompass structural determinants of chromatin compartmentalization.

**TABLE 1:** Aberrant phase separation of PTM-related proteins leading to aging.

Post-translational modification	Chromatin modification/Reader	Phase-separated proteins	Aging-related process/Phenotype	References
H3K4me3 (Active mark)	SGF29 reader	SGF29, MED4, Pol II condensates	Cryptic transcription; cell fate alteration	(Yan et al., 2023)
H3K9me3 (Repressive mark)	HP1α reader	HP1α, KAP1/TRIM28, SIRT3/7	SAHF formation; heterochromatin stabilization	(Bi et al., 2020; Diao et al., 2021; Zhang et al., 2022)
H3K9me3 loss	Lamin B1, APOE	APOE-p62 autophagy pathway	Destabilizing heterochromatin senescence	(Zhao et al., 2022)
H3K27me3 (Repressive mark)	PcG proteins	EZH2, CBX2, CBX7, PRC1	Lifespan regulation; p16INK4a activation	(Uckelmann et al., 2025; Niekamp et al., 2024)
H3K27me3 demethylation	JMJD3/PHF8	JMJD3 IDR condensates	Mitochondrial dysfunction response	(Vicioso-Mantis et al., 2022)
H3K27ac (Active mark)	p300/CBP writer	BRD4-p300 condensates	Gene activation; super-enhancer; Alzheimer’s	(Strom et al., 2024a; Sen et al., 2019)
Deacetylation	HDAC1/3	YY1-METTL3-m6A axis	Premature aging; OIS; telomere shortening	(Li et al., 2022)
γH2AX (DDR)	53BP1 scaffold	53BP1, AHNAK, γH2AX foci	DNA damage repair; p53 activation	(Ghodke et al., 2021; Zhang et al., 2022)
H2Aub	SUMO-RNF168	RNF168 LLPS; SENP1	DNA damage repair; NHEJ efficiency	(Wei et al., 2023)
5-mC	MeCP2 reader	MeCP2, OCT4	Chromatin organization; RTT; AD; AMD	(Wang et al., 2020; Pantier et al., 2024)
Telomeric DNA	Shelterin	TRF1/2 phase separation	Telomere end-protection; DDR	(Jack et al., 2022)

## 3 RNA modification and phase separation in aging

Beyond histone modifications and DNA methylation, emerging evidence has highlighted the critical role of RNA modifications, particularly N6-methyladenosine (m6A), in regulating phase separation and cellular senescence (Wang et al., 2024; Kansal et al., 2026). METTL3, the core component of the m6A methyltransferase complex, possibly recruits the key component of the INO80 chromatin remodeling complex, YY1, to mediate chromatin interactions and chromatin loop formation, thereby shaping 3D genome architecture and suppressing cellular senescence (Chen et al., 2013; Shan et al., 2020; Min et al., 2013). Mechanistically, METTL3 coordinates its enzymatic (m6A methylation) and non-enzymatic (chromatin looping) functions to regulate cellular senescence. During senescence, METTL3-mediated chromatin loops induce HK2 expression, promoting glycolysis and subsequent stress granule phase separation through metabolic reprogramming (Wang et al., 2024) (Figure 1). This establishes a direct link between RNA modification, chromatin organization, LLPS, and aging.

Furthermore, METTL3 depletion leads to increased cellular senescence in multiple cell types, with upregulation of senescence markers including *CDKN1A* (p21) and SASP factors (Chen et al., 2026). The chromatin-associated function of METTL3 is independent of its catalytic activity, indicating a dual-role mechanism wherein METTL3 both writes the m6A epitranscriptomic code and sculpts chromatin architecture to prevent premature aging. In oxidative-stress-induced cellular aging, enhanced METTL3 activity mediates m6A RNA modifications and facilitates the translation of the senescence marker p21, while reduced HDAC2 binding at the p21 promoter further amplifies this effect (Li et al., 2022). METTL3-mediated m6A modification has also been implicated in promoting age-related telomere shortening. These findings collectively suggest that the YY1-HDAC1/3-METTL3 regulatory axis, operating through moderate LLPS, forms a critical nexus linking histone deacetylation, RNA modification, and chromatin phase separation in the aging process. Given the dynamic and reversible nature of both m6A modifications and LLPS, targeting the METTL3 phase separation-chromatin axis represents a promising but yet underexplored avenue for aging intervention. Notably, the role of METTL3 in senescence appears to be context-dependent: its chromatin-architectural, non-catalytic function protects against premature aging (Benayoun et al., 2015), whereas under oxidative stress its catalytic m6A output facilitates p21 translation and telomere attrition (Li et al., 2022). The net effect of METTL3 therefore depends on cell type, stress context, and the balance between these two functions—a duality that warrants direct experimental dissection.

The interplay between RNA modifications and chromatin organization extends beyond METTL3-mediated m6A deposition. RNA itself serves as a critical architectural component of nuclear condensates, with non-coding RNAs actively modulating the biophysical properties of phase-separated compartments. Recent studies have revealed that long non-coding RNAs (lncRNAs) can function as molecular buffers that fine-tune the condensation behavior of prion-like domain (PrLD)-containing proteins, maintaining their functional liquid-like states while preventing pathological solidification (Sun et al., 2024). These findings position RNA not merely as a passive scaffold but as an active rheostat governing condensate material properties across the aging continuum. Together, these advances underscore that RNA modifications and RNA-mediated condensate regulation represent integral layers of epigenetic control that converge with DNA- and histone-based mechanisms to orchestrate chromatin organization during aging.

## 4 DNA modification and phase separation in aging

DNA methylation occurs at the cytosines in CpG dinucleotides to form 5-methylcytosine (5-mC), and 60%–90% of CpG sites in the mammalian genome are methylated. The genome is generally hypomethylated during aging. In contrast, many genes tend to be hypermethylated at CpG islands with aging (Benayoun et al., 2015). MeCP2 is mostly known as a DNA methylation reader and has also been reported as a heterochromatin organizer. MeCP2 was found to tether the SUV39H1-HP1 complex to methylated DNA and was previously shown to accumulate at heterochromatin compartments within the nucleus (Wang et al., 2020). MeCP2 plays a vital role in neuronal development and its malfunction causes various neurodevelopmental disorders. In Alzheimer’s disease (AD), MeCP2 participates in age-related cognitive decline by regulating synaptic plasticity. However, recent studies have challenged the view that MeCP2 contributes to heterochromatin condensates via LLPS, showing instead that MeCP2 binds to methylated DNA independently of phase separation and heterochromatin organisation (Pantier et al., 2024). Quantitative analysis of MeCP2 dynamics revealed a nucleosome-centric model for MeCP2’s dosage sensitivity, with RTT mutations differentially disrupting MeCP2-chromatin interactions (Chua et al., 2024). Cell type-specific 3D-genome studies further demonstrated that MeCP2 facilitates A/B-compartment chromatin segregation, possibly through promoting separate clustering or phase separation of inactive and active chromatin; loss of MeCP2 leads to increased intermixing between active and inactive chromatin, causing transcription mis-regulation (Liu et al., 2025).

Knockdown of MeCP2 impaired synaptic plasticity and cognitive function in senescence accelerated-resistant 1 (SAMR1) mice. Rett syndrome (RTT) is caused by mutations in the X-linked gene encoding MeCP2, these mutations have been reported to compromise MeCP2-mediated chromatin phase separation (Wang et al., 2020). We note that whether MeCP2 organizes heterochromatin through LLPS remains under active debate: other studies indicate that MeCP2 binds methylated DNA independently of phase separation and acts through nucleosome-centric mechanisms (Kansal et al., 2026; Sun et al., 2024; Wang et al., 2020). These views are not mutually exclusive, as DNA binding and condensation may make context-dependent contributions. Spatially resolved mapping of histones in Rett syndrome further revealed selective perturbations in chromatin organization, providing a comprehensive view of how MeCP2 dysfunction affects the epigenome (Schafer et al., 2026). DNA hypomethylation, due to the repression of DNA methyltransferase 1 (DNMT1) and DNMT3A, promotes the cleavage and accumulation of beta-APP in AD and age-related macular degeneration (AMD). Regulation of DNA methylation is necessary for normal development. The loci that display age-dependent DNA hypermethylation are preferentially near tissue-specific genes, genes involved in differentiation and development, genes encoding transcription factors, and transcription factor binding sites (Benayoun et al., 2015). Studies in stem cells have demonstrated that loci with age-associated DNA hypermethylation are enriched for genes normally repressed by PcG that methylate H3K27 (Teschendorff et al., 2010), suggesting that histone-modifying enzymes result in aberrant recruitment of the DNA methylation enzymes during aging. Notably, recent work has proposed that somatic mutations may be a primary driver of epigenetic aging (DNA methylation clocks), challenging the view that methylation changes directly cause aging (Koch et al., 2025), while population-specific DNA methylation clocks continue to demonstrate the interplay between epigenetic marks and chronological age (Zheng et al., 2024).

Beyond MeCP2-mediated chromatin organization, architectural proteins that coordinate higher-order chromatin structure play pivotal roles in aging-associated transcriptional programs. High mobility group protein A1 (HMGA1) has recently been identified as a key organizer of 3D chromatin networks through its heterogeneous linear distribution along the genome (Olan et al., 2024). HMGA1-dense genomic loci form highly interconnected chromatin interaction networks that function as transcriptional buffers against inflammatory signaling. In the context of cellular senescence, HMGA1 deficiency leads to further upregulation of SASP genes, promoting aging heterogeneity across cell populations. These findings establish HMGA1 as a critical chromatin architectural factor whose phase separation-mediated network organization constrains inflammatory gene expression during aging, bridging DNA-level chromatin compartmentalization with the macroscopic phenotype of senescence-associated inflammation. The integration of HMGA1-mediated chromatin buffering with established DNA methylation dynamics reveals a multi-layered regulatory architecture wherein sequence-level epigenetic marks and structural chromatin organizers cooperate to maintain transcriptional homeostasis during aging.

The establishment and maintenance of 3D chromatin architecture also depend on the coordinated action of structural maintenance of chromosomes (SMC) complexes and transcriptional regulators. Recent work in fission yeast has uncovered a functional interaction between the condensin subunit Cnd1 and the mediator subunit Pmc4, revealing that condensin-mediator cooperation contributes to mitotic chromatin domain formation (Iwasaki et al., 2026). A cnd1-K658E mutation that disrupts this interaction impairs condensin-mediated chromatin domain assembly during mitosis, while 1,6-hexanediol treatment and Pmc4 depletion compromise both mitotic gene expression and condensate boundary integrity. These findings demonstrate that Mediator contributes to gene expression and chromosome architecture through phase separation principles, extending the functional repertoire of transcriptional condensates beyond interphase chromatin organization to cell cycle-dependent genome regulation. The conservation of such mechanisms in higher eukaryotes suggests that condensin-mediator condensates may represent evolutionarily ancient modules for coupling transcriptional control with chromatin structural dynamics.

The ectopic expression of pluripotent factor OCT4 restored young DNA methylation patterns and transcriptome, thereby reversing visual loss in aging mice. It has been demonstrated that phase separation ability of OCT4 contributes to the cell fate transitions during reprogramming by regulating chromatin 3D structure, such as TAD reorganization and chromatin compartmentalization (Wang et al., 2021). Collectively, these observations support the broader hypothesis that phase separation regulates aging-related genes through chromatin structural factors, and that remodeling of such structures may be a prerequisite for cell fate transitions. Recent studies have further shown that transcriptional condensates form in a stepwise manner to enable graded and 3D gene control, promoting adaptive 3D genome reconfiguration beyond simple gene activation (Chowdhary et al., 2026), while selective phase separation of transcription factors is driven by orthogonal molecular grammar that determines condensate composition and function (Driver and Onck, 2025). Conversely, transcriptional activity itself can organize euchromatin through microphase separation, underscoring the bidirectional coupling between transcriptional condensates and chromatin architecture (Hilbert et al., 2021). Importantly, whether condensate formation *per se* enhances transcription remains under debate: multivalent activation-domain interactions can enhance transcription independently of droplet formation, and forced transcription-factor droplets can have a neutral or even inhibitory effect on activation (Trojanowski et al., 2022).

Eukaryotic chromosomes end with telomeres to protect the genome during cell divisions. In somatic cells, telomere shortening is a major feature of replicative senescence (Maciejowski and de Lange, 2017). Shelterin proteins, such as TRF1/2, can phase separate with telomeric DNA to build telomere compartments, and these liquid droplets selectively recruit telomere-associated factors (Jack et al., 2022). Recent studies have revealed that TERRA (telomeric repeat-containing RNA) forms phase-separated condensates with LSD1 through G-quadruplex interactions, enriching Rad51AP1 and increasing TERRA-containing R-loops at telomeres to maintain telomere stability (Xu et al., 2024). Additionally, hnRNPA1 and TERRA phase separation has been shown to regulate telomeric stability, with nucleic acids significantly increasing hnRNPA1 phase-separating ability (Xu et al., 2025). SUMOylation-driven phase separation at telomeres independently of PML bodies has also been demonstrated, with SUMO-induced condensates forming through co-phase separation of DNA repair factors (Zhao et al., 2024). TERRA and telomeric damage-induced long ncRNAs (tdilncRNA) are transcribed at telomeres and are known to regulate telomere length. Expression of TERRA is upregulated by telomere H3K9me3 and HP1 proteins deficiency (Aguado et al., 2019), thereby promoting aging through activating DDR at telomeres.

Collectively, these findings illustrate a comprehensive model of how phase separation-mediated chromatin compartmentalization undergoes spatiotemporal reorganization during cellular senescence (Table 2). In young cells, LLPS orchestrates precise 3D chromatin architecture through constitutive heterochromatin liquid droplets (H3K9me3-HP1-IRTKS), facultative heterochromatin (H3K27me3-PRC1), and active euchromatin transcriptional hubs buffered by architectural proteins such as HMGA1 (Figure 2). During aging, this architecture erodes through multiple convergent mechanisms: depletion of stabilizing marks (PRMT1-dependent H4R3me2as degradation) leads to nucleosome instability and heterochromatin dissolution; aberrant super-enhancer condensates (H3K27ac-BRD4/p300) hyperactivate SASP genes; pathological FUS phase transitions impair CTCF binding to induce TAD fusion; and nucleolar expansion acts as a biomolecular mortality timer (detailed in the section “Nucleolar Condensates as Aging Timer”; Figures 2, 3A). These multi-layered changes collectively transform the nuclear landscape from an organized, functional compartmentalized state to a dysregulated, senescent chromatin environment.

**TABLE 2:** Phase separation regulators of chromatin organization in cellular senescence.

Regulator	Epigenetic mark/Function	Condensate mechanism	Role in senescence	References
HP1α	H3K9me3 reader	Multivalent PTM-reader interaction; LLPS with KAP1/SIRT3/7	SAHF formation; heterochromatin stabilization counteracts senescence	(Wang et al., 2019; Williams et al., 2024)
PRC1-CBX2/CBX7/CBX8	H3K27me3 reader; PcG protein	Phase-separated condensates at H3K27me3 loci	Lifespan regulation; p16INK4a repression porous chromatin condensate structure	(Uckelmann et al., 2025; Niekamp et al., 2024)
EZH2	H3K27me3 methyltransferase	PRC2 complex phase separation	Heterochromatin formation; lifespan regulation	(Uckelmann et al., 2025)
JMJD3	H3K27me3 demethylase	IDR-driven condensate formation at enhancer clusters	Positive lifespan regulator; mitochondrial dysfunction response	(Vicioso-Mantis et al., 2022)
BRD4-p300/CBP	H3K27ac reader/writer	Acetylation-driven immiscible phase separation	Super-enhancer formation; gene activation; drives senescence phenotype	(Strom et al., 2024a; Sen et al., 2019)
YY1-HDAC1/3-METTL3	Histone deacetylation; m6A writer	Moderate LLPS of YY1-METTL3 axis	Premature aging; OIS; telomere shortening p21 translation	(Li et al., 2022)
53BP1-AHNAK	γH2AX scaffold; DDR protein	Oligomerization-driven LLPS at DSB foci	DNA damage repair; p53 activation heterochromatin maintenance	(Ghodke et al., 2021; Zhang et al., 2022)
RNF168-SENP1	H2A ubiquitin ligase; SUMO protease	SUMOylation-regulated LLPS at DNA damage sites	NHEJ repair efficiency; DNA damage response	(Wei et al., 2023)
MeCP2	5-mC reader	Chromatin phase separation; tethering SUV39H1-HP1 complex	Chromatin organization; neuronal development; RTT; AD; AMD	(Wang et al., 2020; Pantier et al., 2024)
SGF29-MED4-Pol II	H3K4me2/3 reader; co-activator	Transcriptional condensate formation	Cryptic transcription; cell fate alteration cell senescence gene activation	(Yan et al., 2023)
HMGA1	Architectural chromatin protein	Heterogeneous linear genome distribution; 3D network formation	Transcriptional buffer for inflammatory signaling; SASP gene regulation	(Olan et al., 2024)
IRTKS	Heterochromatin remodeling factor	Senescence-associated condensation drives heterochromatin rearrangement	Counteracts cellular senescence; endothelial function	(Xie et al., 2024)
FUS	RNA-binding protein; RBP	Aberrant phase transition; C-terminal RGG repeat-driven LLPS	TAD fusion; CTCF binding impairment; HSC aging; DDR initiation	(Tang et al., 2024; Levone et al., 2021)
OCT4	Pluripotency transcription factor	Phase separation drives TAD reorganization and chromatin compartmentalization	Reprogramming; cell fate transitions reversal of aging phenotypes	(Wang et al., 2021)
TRF1/2	Shelterin complex; telomere binding	DNA-scaffolded phase separation with telomeric DNA	Telomere end-protection; telomere-associated factor recruitment; DDR	(Jack et al., 2022)

**FIGURE 2:**
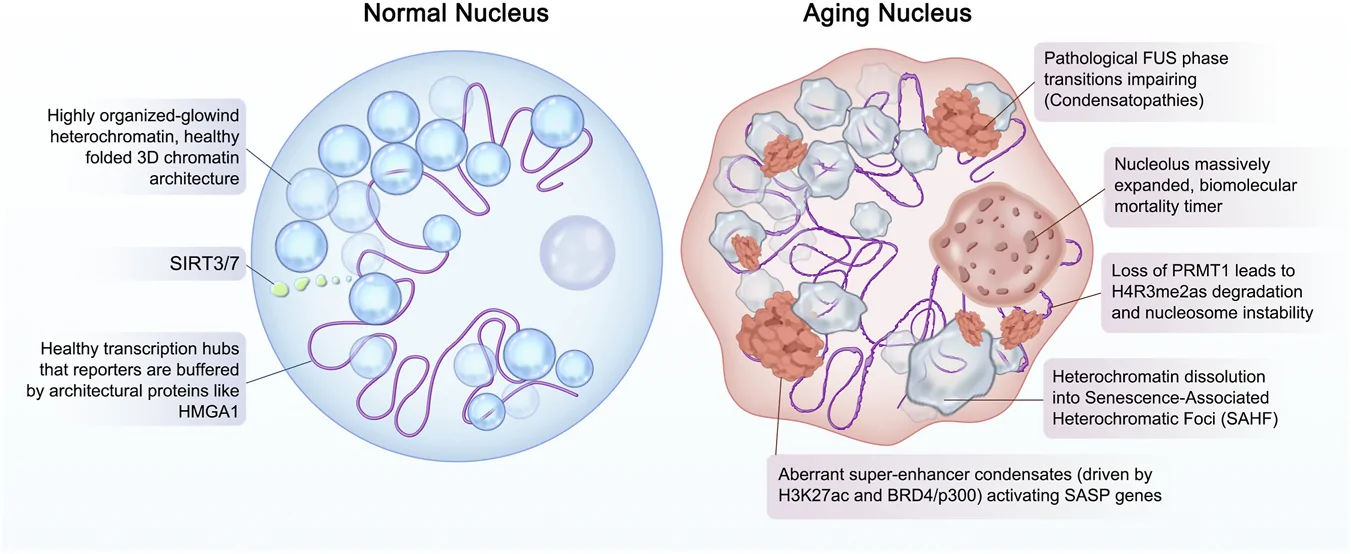
Spatiotemporal reorganization of chromatin compartmentalization mediated by phase separation during cellular senescence. Left: Epigenetic Compartmentalization in Young Cells. In normal physiological states, liquid-liquid phase separation (LLPS) strictly orchestrates precise 3D chromatin architecture. Constitutive heterochromatin forms liquid-like droplets via multivalent interactions among specific histone marks (e.g., H3K9me3) and readers (HP1, IRTKS), stabilized at the nuclear lamina by SIRT3/7. Active euchromatin maintains delicate transcriptional hubs buffered by architectural proteins like HMGA1, ensuring healthy cellular identity. Right: Aberrant Condensate Dynamics in Senescent Cells. During aging, the global epigenetic landscape undergoes massive structural erosion. Depletion of stabilizing marks (e.g., PRMT1-dependent H4R3me2as degradation) leads to nucleosome instability and heterochromatin dissolution, culminating in Senescence-Associated Heterochromatic Foci (SAHF). Concurrently, aberrant super-enhancer condensates (driven by H3K27ac and BRD4/p300) hyperactivate Senescence-Associated Secretory Phenotype (SASP) genes. Furthermore, pathological FUS phase transitions impair CTCF binding to induce Topologically Associating Domain (TAD) fusion, while the nucleolus massively expands, acting as a biomolecular mortality timer.

**FIGURE 3:**
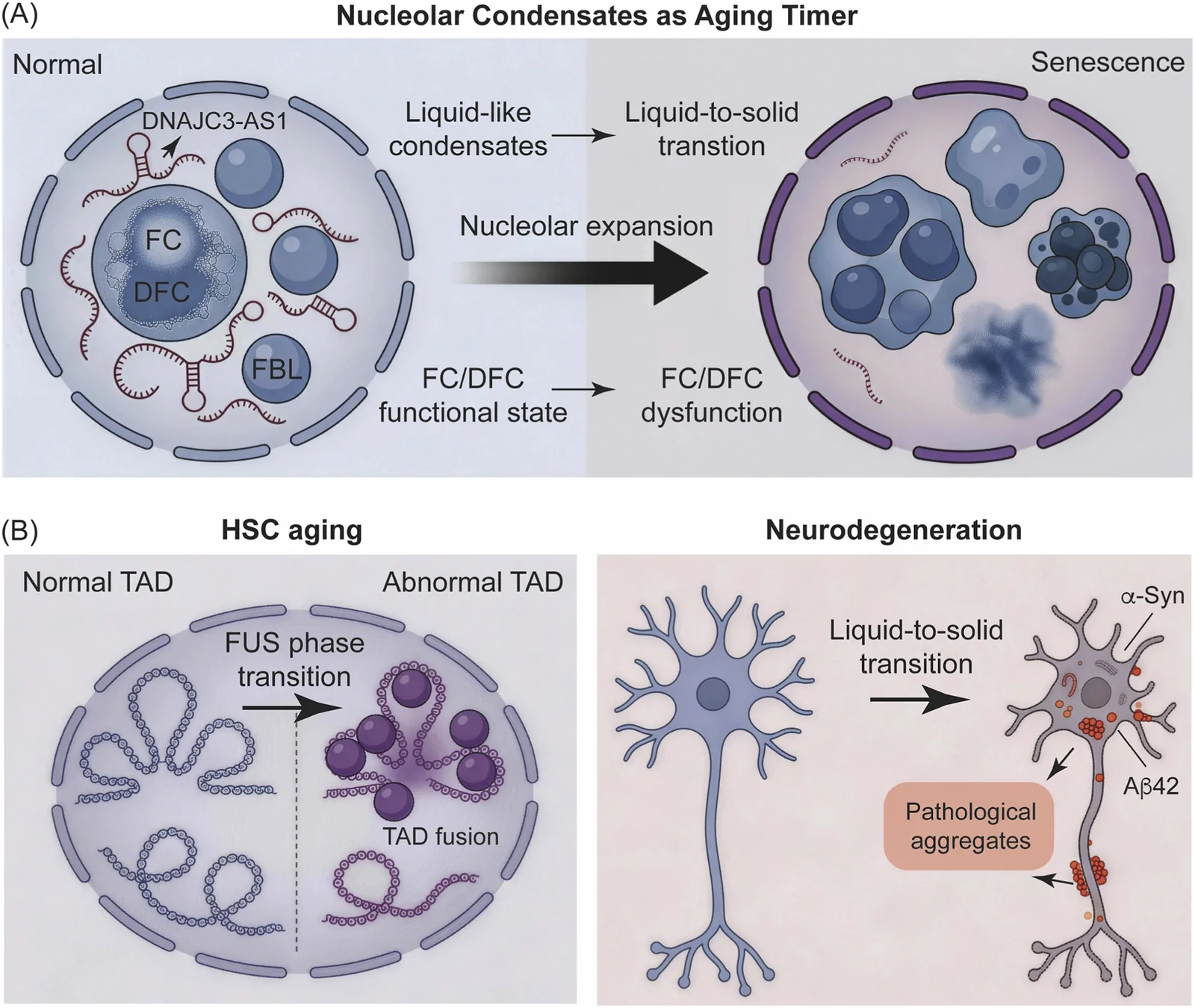
Phase separation dysregulation in nucleolar aging and age-related diseases. **(A)** Nucleolar condensate expansion as a biomolecular timer of cellular aging: lncRNA DNAJC3-AS1 buffers FBL condensation to maintain FC/DFC functional states and prevent pathological liquid-to-solid transitions. **(B)** Phase separation dysregulation in stem cell aging and neurodegeneration: FUS phase transition impairs HSC function via TAD fusion; alpha-synuclein and Aβ42 undergo pathological phase transitions driving Parkinson’s and Alzheimer’s disease pathology through cross-protein co-condensation and lipid-induced aggregation.

## 5 Nucleolar condensates as aging timer

Recent compelling evidence has positioned the nucleolus as a key biomolecular condensate whose biophysical properties act as a mortality timer during aging. Studies in yeast have demonstrated that nucleolar expansion beyond a critical size threshold triggers a mortality timer, during which the biophysical properties of nucleolar condensates change, allowing entry of repair proteins and triggering rDNA instability (Gutierrez and Tyler, 2024; Boukoura and Larsen, 2024) (Figure 3A). In human cells, nucleolar decondensation during senescence leads to increased chromatin accessibility and SASP expression, linking nucleolar condensate integrity to chromatin state changes and inflammatory gene expression (Lopes-Paciencia and Ferbeyre, 2025). The hierarchical interactions between nucleolar and heterochromatin condensates, mediated by dual-affinity proteins such as nucleolin, further reveal how different nuclear condensates coordinate to maintain genome stability and how their dysregulation contributes to aging (Rajshekar et al., 2025). These findings establish nucleolar phase separation dynamics as both a biomarker and mechanistic driver of cellular aging.

A recent study illuminated how non-coding RNAs actively govern nucleolar condensate homeostasis through sophisticated biophysical mechanisms. The long non-coding RNA DNAJC3-AS1 has been identified as a critical rheostat that buffers the condensation behavior of fibrillarin (FBL), a key nucleolar protein containing prion-like domains (PrLDs), thereby maintaining the functional integrity of the fibrillar center (FC) and dense fibrillar component (DFC) unit (Sun et al., 2024). This lncRNA simultaneously promotes FBL condensation and inhibits excessive aggregation, effectively preventing the pathological liquid-to-solid transition that would otherwise compromise ribosomal RNA processing and nucleolar function. By revealing that lncRNAs maintain the functional phase state of prion-like proteins through molecular buffering, this study establishes a paradigm for understanding how RNA-protein condensate crosstalk preserves organelle identity and function during aging. The dysregulation of such lncRNA-mediated buffering mechanisms may contribute to age-dependent nucleolar dysfunction and the acceleration of cellular senescence programs.

## 6 Closing discussion

At present, whether phase separation regulates cell senescence has attracted great attention, as both are closely linked to post-translational modifications, and chromatin 3D structure, especially in the synergistic effect of the heterochromatin compartment assembly (Xie and Han, 2025). Additionally, due to the dynamic and reversible nature of LLPS, precise regulation of epigenetics will be a promising intervention for slowing down the aging process (Li et al., 2026; Qin et al., 2025; Fleming and Schuijers, 2026). The concept of “condensatopathies” has recently emerged as a unified mechanistic framework for diseases arising from dysregulated biomolecular condensates, classifying mechanisms as Loss-of-Function (failure to form condensates) and Toxic Gain-of-Function (aberrant condensates), and proposing integrated theranostic intervention strategies (Li et al., 2026). This framework is particularly relevant to aging, as age-dependent decline in stress granule formation and other condensate functions contributes to age-related cellular vulnerability.

The convergence of Loss-of-Function (LOF) and Toxic Gain-of-Function (TGOF) mechanisms in aging-related condensatopathies provides a unified conceptual framework for understanding how dysregulated phase separation contributes to disease (Table 3). LOF mechanisms, exemplified by SIRT3/7 deficiency leading to heterochromatin disassembly, IRTKS loss driving SASP activation, and PRMT1-mediated H4R3me2as reduction causing nucleosome instability, share the common feature of condensate failure that compromises chromatin integrity. Conversely, TGOF mechanisms, including alpha-synuclein (αSyn) aggregation in Parkinson’s disease, Αβ42 amyloid formation in Alzheimer’s disease, and FUS-mediated TAD fusion in HSC aging, represent pathological solidification of normally dynamic condensates. Nucleolar expansion represents a unique category where both LOF (functional decline) and TGOF (material property changes) operate in concert to drive the mortality timer.

**TABLE 3:** Condensatopathy classification in aging-related diseases.

Category	Mechanism	Representative protein	Disease/Model	Intervention strategy	References
LOF	SIRT3/7 deficiency → heterochromatin disassembly	SIRT3/7	hMSC aging stem cell senescence	SIRT3/7 overexpression; NAD + boosters	(Bi et al., 2020; Diao et al., 2021)
LOF	IRTKS loss → SASP activation heterochromatin rearrangement	IRTKS	Endothelial senescence; OIS	IRTKS condensate stabilization	(Xie et al., 2024)
LOF	PRMT1↓ → H4R3me2as↓ → nucleosome disassembly	PRMT1	Cellular senescence	Metformin; rapamycin resveratrol	(Lin et al., 2020)
LOF	MRG15 depletion → accelerated senescence	MRG15	hMSC aging	MRG15 condensate promotion	(Li et al., 2025)
LOF	ERG phase separation ↓ → senescence gene expression ↑	ERG	Endothelial cell aging	ERG condensate stabilization	(Pu et al., 2026)
LOF	Nucleolar functional decline (rRNA processing ↓)	FBL< (fibrillarin)	Cellular senescence mortality timer	lncRNA-mediated buffering (DNAJC3-AS1)	(Sun et al., 2024)
TGOF	α-synuclein aberrant aggregation Lewy bodies	α-synuclein	Parkinson’s disease	Key motif deletion phase separation modulators	(Tang et al., 2025)
TGOF	Aβ42 amyloid aggregation nucleation for αSyn	Aβ42	Alzheimer’s disease	Lipid bilayer interference aggregation inhibitors	(Röntgen et al., 2025; Šneiderie et al., 2025)
TGOF	FUS aberrant condensates → TAD fusion	FUS	HSC aging; hematopoietic aging	FUS phase transition modulators	(Tang et al., 2024)
TGOF	SGF29 condensate formation → cryptic transcription	SGF29	Stem cell aging	SGF29 condensate dissolution; H3K4me3 modulation	(Yan et al., 2023)
TGOF	p300/CBP-driven super-enhancer → senescence	p300/CBP	Replicative senescence; AD	p300 depletion; HAT inhibitors	(Sen et al., 2019)
Both (LOF TGOF)	Nucleolar expansion functional decline + material property changes → mortality timer	Nucleolin (dual-affinity protein)	Yeast aging; mammalian senescence	Nucleolar size monitoring Condensate property modulation	(Rajshekar et al., 2025; Gutierrez and Tyler, 2024)

Furthermore, transcription factors such as ERG have been identified as longevity-associated regulators that form nuclear condensates through LLPS, altering chromatin organization and reducing senescence gene expression to attenuate cellular senescence (Pu et al., 2026), while super-enhancer-driven core transcription factor FOXP1 delays endothelial cell senescence via phase separation-mediated SESN3 activation (Mao et al., 2026).

The convergence of phase separation dysregulation and aging extends prominently into stem cell biology. In hematopoietic stem cells (HSCs), age-dependent accumulation of the RNA-binding protein FUS leads to the formation of aberrant FUS condensates that impair HSC mobility and regenerative capacity (Tang et al., 2024) (Figure 3B). This study provides the first evidence that FUS phase transition promotes HSC aging through chromatin structural remodeling, establishing a direct causal link between RNA-binding protein phase separation, 3D genome reorganization, and stem cell exhaustion. Given the central role of HSCs in organismal aging and tissue homeostasis, FUS-mediated condensatopathy in the hematopoietic system may represent a paradigm for understanding how phase separation defects in tissue-specific stem cells propagate aging phenotypes at the organismal level.

The principles of phase separation-mediated chromatin dysregulation also find striking parallels in neurodegenerative diseases, where the pathological conversion of functional condensates into aggregates represents a unifying disease mechanism. In Parkinson’s disease (Hauser et al., 2017), αSyn undergoes phase separation through seven beta-sheet-enriched motifs that drive its condensation (Tang et al., 2025). Two C-terminal motifs form a percolated network through transient hydrogen bonds, and deletion of key motifs reduces phase separation capacity, revealing the specific sequence determinants that govern αSyn condensate formation in early PD pathogenesis. In Alzheimer’s disease (AD), amyloid-beta 42 (Αβ42) forms large aggregates that serve as heterogeneous nucleation sites to initiate αSyn phase separation, while Aβ40 is sequestered into αSyn condensates where it accelerates the liquid-to-solid transition (Röntgen et al., 2025). This cross-talk between AD and PD pathological proteins through co-condensation highlights a shared mechanistic basis for protein aggregation disorders. Furthermore, Αβ42 has been shown to undergo liquid-liquid phase separation on lipid bilayers, forming intrinsically heterogeneous condensates that contain phospholipids and subsequently undergo liquid-to-solid transition to form amyloid fibrils (Šneiderie et al., 2025). These lipid-driven phase transitions represent critical early steps in Αβ42 fibrillogenesis, linking membrane biology with condensate-mediated protein aggregation. Collectively, these findings illuminate how the dysregulation of phase separation transitions—from liquid to solid states—serves as a convergent pathogenic mechanism in both aging and neurodegeneration, reinforcing the concept of condensatopathies as a framework for understanding age-related protein aggregation diseases.

These diverse condensatopathy mechanisms can be unified under a common mechanistic paradigm of biomolecular condensate maturation and pathological phase transitions. In healthy states, lncRNAs such as DNAJC3-AS1 act as molecular rheostats to buffer the phase states of prion-like domain proteins such as fibrillarin, maintaining functional nucleolar condensates. METTL3 shapes healthy chromatin contacts and drives m6A-mediated stress granule formation to ensure cellular homeostasis (Figure 4A). However, aging impairs these buffering capacities, triggering pathological liquid-to-solid transitions. In aging hematopoietic stem cells, elevated FUS forms solid-like aberrant condensates that disrupt CTCF binding and induce higher-order 3D genomic fusion, providing a direct causal link between RBP phase transition and stem cell exhaustion (Figure 4B). Furthermore, the condensatopathy framework bridges organismal aging and neurodegeneration: intrinsically disordered proteins such as αSyn and Αβ42 initially undergo dynamic LLPS, but aging-associated stresses and lipid bilayer interactions trigger heterogeneous cross-seeding and liquid-to-solid transition, accelerating toxic amyloid fibril formation in Parkinson’s and Alzheimer’s diseases (Figure 4C). This unified framework positions condensate material properties as both biomarkers and therapeutic targets across the aging spectrum.

**FIGURE 4:**
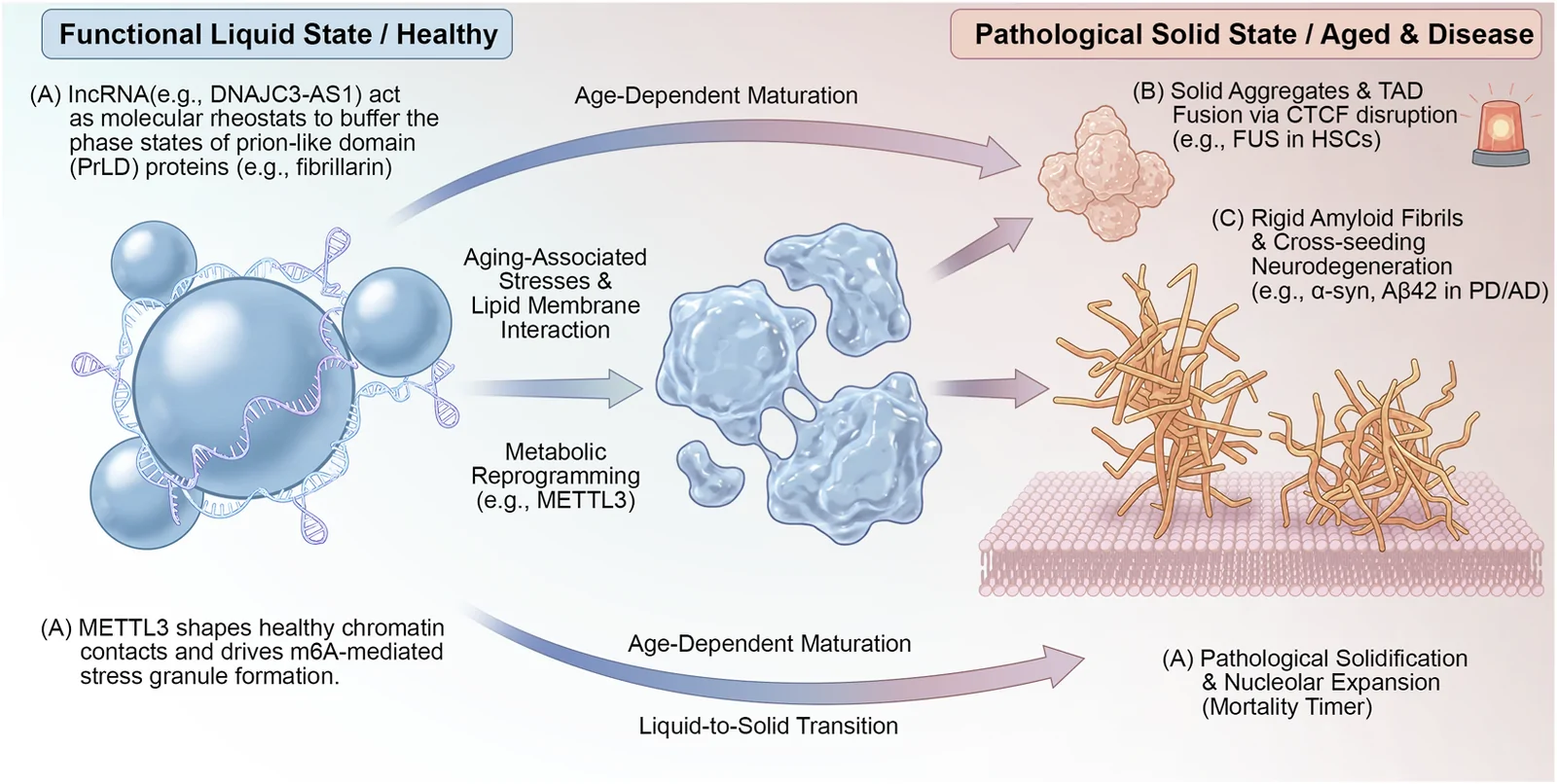
Mechanistic paradigms of condensatopathies: Biomolecular condensate maturation and pathological phase transitions. Biomolecular condensates undergo age-dependent maturation characterized by altered diffusion dynamics and liquid-to-solid phase transitions. **(A)** The Nucleolar Mortality Timer and RNA Buffering: In healthy states, lncRNAs (e.g., DNAJC3-AS1) act as molecular rheostats to buffer the phase states of prion-like domain (PrLD) proteins (e.g., fibrillarin). Aging impairs this buffering capacity, triggering a pathological liquid-to-solid transition and nucleolar expansion. Similarly, METTL3 shapes healthy chromatin contacts and drives m6A-mediated stress granule formation. **(B)** Stem Cell Exhaustion and Chromatin Collapse: In aging hematopoietic stem cells (HSCs), the accumulation of RNA-binding proteins like FUS leads to the formation of solid-like aberrant condensates that disrupt CTCF binding, inducing higher-order 3D genomic fusion. **(C)** Neurodegenerative Co-condensation: The condensatopathy framework bridges organismal aging and neurodegeneration. Intrinsically disordered proteins, such as alpha-synuclein and Amyloid-beta (Aβ42), initially undergo dynamic LLPS. However, aging-associated stresses and lipid bilayer interactions trigger heterogeneous cross-seeding and a liquid-to-solid transition, accelerating the formation of toxic, rigid amyloid fibrils in Parkinson’s and Alzheimer’s diseases. Abbreviations used in this figure: PrLD, prion-like domain; FBL, fibrillarin; FC/DFC, fibrillar center/dense fibrillar component; m6A, N6-methyladenosine; HSC, hematopoietic stem cell; TAD, topologically associating domain; Aβ42, amyloid-beta 42.

Across these diverse biological contexts—chromatin compartmentalization in senescent somatic cells, stem cell exhaustion, and neurodegenerative protein aggregation—phase separation emerges as a fundamental biophysical principle governing cellular homeostasis and its age-dependent decline. The identification of molecular determinants that control condensate phase states, from lncRNA-mediated buffering of nucleolar proteins to lipid surface-induced amyloid phase transitions, provides multiple entry points for therapeutic intervention. Future efforts to modulate condensate material properties, rather than simply dissolving or preventing their formation, hold particular promise for developing precision therapeutics that can restore functional condensate dynamics while preserving their essential physiological roles. An integrated understanding of how phase separation orchestrates chromatin architecture, RNA metabolism, and protein homeostasis across the lifespan will be essential for translating the condensatopathy framework into effective anti-aging strategies.

The integration of biomolecular condensate biology with established aging hallmarks reveals multiple entry points for therapeutic intervention (Table 4). From 53BP1-HP1α condensates that safeguard genomic stability, to PRC1-CBX condensates that maintain epigenetic fidelity, to TERRA-LSD1 phase separation that protects telomere integrity, each aging hallmark can now be mapped onto specific condensate systems (Xu et al., 2024). The therapeutic implications are substantial: rather than targeting individual genes or pathways, modulating condensate material properties—enhancing formation where LOF drives pathology, dissolving aberrant aggregates where TGOF is causal, and buffering transitions where condensate aging compromises function—represents a paradigm shift in anti-aging drug development. The “See-and-Treat” approach, combined with emerging condensate-modifying drugs (c-mods) that operate beyond simple dissolution, positions the condensatopathy framework as a transformative lens for translating aging biology into precision therapeutics.

**TABLE 4:** Biomolecular condensates across aging hallmarks and therapeutic opportunities.

Aging hallmark	Condensate/System	Mechanistic link	Therapeutic target	References
Genomic instability	53BP1-HP1α condensates	LLPS maintains heterochromatin integrity; 53BP1 scaffold for DNA repair foci	53BP1 oligomerization modulators; AHNAK-based regulation	(Ghodke et al., 2021; Zhang et al., 2022)
Epigenetic alterations	PRC1-CBX condensates; HP1α condensates	H3K27me3/H3K9me3 compartment formation via multivalent reader interactions	PRC1 modularity targeting; HP1α stabilization	(Uckelmann et al., 2025; Niekamp et al., 2024)
Telomere attrition	TERRA-LSD1; TRF1/2-shelterin; hnRNPA1	Phase separation compartmentalizes telomere ends; G-quadruplex-driven condensates	TERRA-LSD1 interaction modulators shelterin stabilization	(Jack et al., 2022; Xu et al., 2024)
Mitochondrial dysfunction	JMJD3 IDR condensates	H3K27me3 demethylation at enhancers responds to mitochondrial stress	JMJD3 condensate formation modulators	(Vicioso-Mantis et al., 2022)
Cellular senescence	IRTKS condensates; HMGA1 compartments	Heterochromatin remodeling transcriptional buffering of inflammatory signals	IRTKS condensate promotion; HMGA1 expression restoration	(Olan et al., 2024; Xie et al., 2024)
Stem cell exhaustion	FUS condensates; OCT4 phase separation	FUS TAD fusion and chromatin architecture disruption; OCT4 reprogramming	FUS phase transition correction; OCT4 condensate promotion	(Tang et al., 2024; Wang et al., 2021)
Loss of proteostasis	Nucleolar condensates (FBL, PrLD proteins)	lncRNA-mediated buffering of condensate phase states mortality timer	lncRNA-based therapeutics nucleolar size intervention	(Sun et al., 2024; Gutierrez and Tyler, 2024)
Neurodegenerative aggregation	α-synuclein; Aβ42 condensates	Pathological liquid-to-solid transition; crossprotein cocondensation	Phase separation modulators (c-mods); motif-targeting peptides	(Tang et al., 2025; Röntgen et al., 2025)
Chronic inflammation	SASP-IRTKS-HMGA1 axis; p300 super-enhancers	Chromatin compartmentalization buffers inflammatory signaling enhancer-driven SASP expression	HMGA1 pathway activation; p300 inhibition chromatin remodelers	(Olan et al., 2024; Sen et al., 2019; Xie et al., 2024)
RNA modification dysregulation	YY1-HDAC1/3-METTL3-m6A axis	Moderate LLPS links histone diacetylation to RNA modification and chromatin architecture	METTL3 condensate modulation; HDAC1/3 activity regulators	(Li et al., 2022)
